# Emerging Pathway to a Precision Medicine Approach for Angina With Nonobstructive Coronary Arteries in Women

**DOI:** 10.1016/j.jacadv.2024.101074

**Published:** 2024-07-04

**Authors:** Nisha Hosadurg, Kelsey Watts, Shuo Wang, Kelly E. Wingerter, Angela M. Taylor, Todd C. Villines, Amit R. Patel, Jamieson M. Bourque, Jonathan R. Lindner, Christopher M. Kramer, Garima Sharma, Patricia F. Rodriguez Lozano

**Affiliations:** a Cardiovascular Division, Department of Medicine, University of Virginia Health, Charlottesville, Virginia, USA; b Center for Public Health Genomics, University of Virginia School of Medicine, Charlottesville, Virginia, USA; c INOVA Heart and Vascular Institute, Fairfax, Virginia, USA; d Department of Radiology and Medical Imaging, University of Virginia Health, Charlottesville, Virginia, USA

**Keywords:** coronary microvascular dysfunction, endothelial dysfunction, epigenetics, patient-centered care, social determinants of health, vasospasm

## Abstract

Women are disproportionately affected by symptoms of angina with nonobstructive coronary arteries (ANOCA) which is associated with significant mortality and economic impact. Although distinct endotypes of ANOCA have been defined, it is underdiagnosed and is often incompletely characterized when identified. Patients are often unresponsive to traditional therapeutic options, which are typically antianginal, and the current ability to guide treatment modification by specific pathways is limited. Studies have associated specific genetic loci, transcriptomic features, and biomarkers with ANOCA. Such panomic data, in combination with known imaging and invasive diagnostic techniques, should be utilized to define more precise pathophysiologic subtypes of ANOCA in women, which will in turn help to identify targeted, effective therapies. A precision medicine-based approach to managing ANOCA incorporating these techniques in women has the potential to significantly improve their clinical care.

## Highlights

• ANOCA is highly prevalent, disproportionately affects women, and exhibits significant pathophysiologic and phenotypic heterogeneity, resulting in subtherapeutic responses to traditional antianginals with persistent symptoms.
• Specific pathophysiologic endotypes, gene variants, proteomic biomarkers, and radiologic features associated with specific mechanisms and prognosis in ANOCA have been identified, however it remains to be seen whether these discoveries can improve patient symptoms and outcomes by being incorporated into decision-making.
• A precision medicine-based approach toward defining endophenotypes incorporating panomic data to enable targeted therapies for women suffering from ANOCA is a critical need.

Angina with nonobstructive coronary arteries (ANOCA) has a prevalence of ∼50% in patients undergoing ischemia testing, is highly prevalent in women, and has a significant propensity for major adverse cardiovascular events (MACE).^1^ ANOCA is a heterogeneous entity with multiple mechanistic pathways or endotypes^1^ associated with a variety of genetic, epigenetic, and inflammatory mechanisms. ANOCA also has differential gender/social determinants leading to complex phenotypic expression. Currently, traditional antianginal drugs such as beta-blockers, calcium-channel blockers (CCBs), and nitrates remain the mainstay of treatment, yet are modestly effective in this population. A key issue has been the heterogeneous pathobiology of ANOCA patients studied and a lack of endotype-specific trials. Precision medicine for ANOCA, both from diagnostic and therapeutic standpoints, has the potential to improve both symptoms and outcomes.

In this article, we review the pathophysiologic mechanisms of ANOCA endotypes, the specific genetic, epigenetic, inflammatory pathways, stratified diagnostic approaches, and precision medicine-driven treatment strategies (outlined in the Central Illustration), within a multidisciplinary, patient-centered team.

**Central Illustration:**
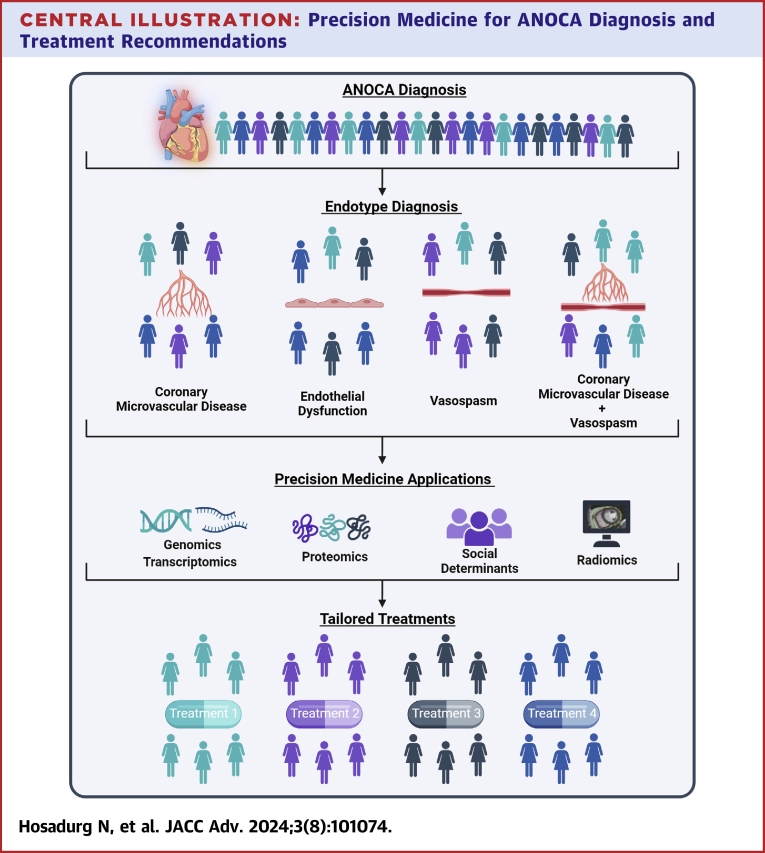
**Precision Medicine for ANOCA Diagnosis and Treatment Recommendations** ANOCA = angina with nonobstructive coronary arteries.

## Epidemiology

The standardized term ANOCA, describing a broad range of symptoms concerning for ischemia in the absence of identifiable angiographic obstructive coronary disease, only evolved recently.^2^ However, limited forms of this entity were described as early as 1973 as cardiac syndrome X. In this review, we use the term ANOCA rather than INOCA (ischemia with nonobstructive coronary arteries) since the confirmation of ischemia is not necessarily a criterion for a working diagnosis of ANOCA.^3^ Furthermore, traditional invasive or noninvasive methods may not be able to definitively rule out ischemia caused by the variety of nonobstructive causes that contribute to this diagnosis.

ANOCA is associated with distinct pathophysiologic endotypes, including coronary microvascular dysfunction (CMD), endothelial dysfunction, epicardial, and microvascular vasospasm. A recent meta-analysis of the prevalence of ANOCA endotypes in ∼6,500 patients reported that CMD alone occurred in 23% of patients, epicardial coronary spasm in 40%, microvascular spasm in 24%, and CMD and coronary spasm together occurred in 23% of patients. These differences in endotype prevalence alone reflect significant heterogeneity in vasomotor abnormalities that necessitate precision medicine-based assessment. This study also reported that women were 1.45 times more likely to have CMD than men.^4^ Despite recent work, data on distinct mechanisms underlying these endotypes and associated prognostic implications are lacking.

Both sex- and gender-specific factors can contribute to the heterogeneity of ANOCA and should be considered when evaluating and managing these patients. The biological influences of sex include those exerted by genetic differences due to the XX (female) and XY (male) chromosomes, sex hormones, age, and pregnancy history.^5^ Gender influences include access to care, employment, cost of drugs, culture, and lifestyle factors. For this review, we used the terms females/males or women/men in a manner that maintained in consistency in the terminology with the original source referenced.

**Figure 1:**
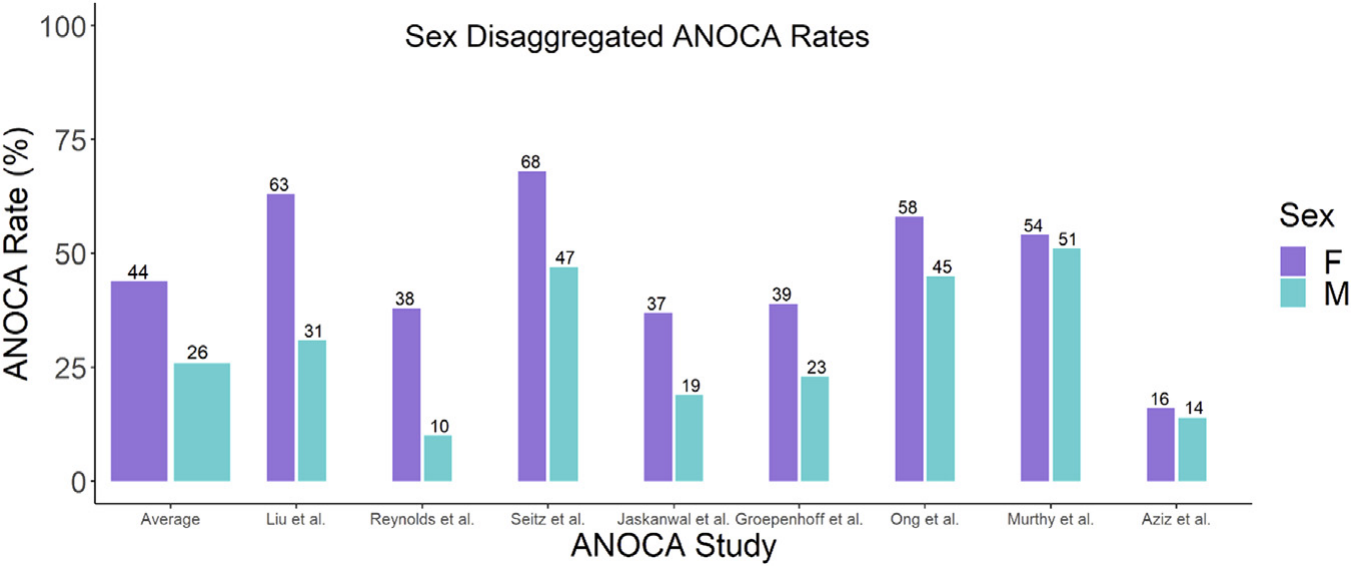
**Sex-Specific Prevalence of ANOCA** Sex-stratified rates of ANOCA diagnosis in men and women from large cohort studies of patients presenting with ischemia. ANOCA = angina with nonobstructive coronary arteries.

Many studies have established the female sex-biased rates of ANOCA. The WISE (Women’s Ischemia Syndrome Evaluation) study reported that among the ∼500,000 U.S. women undergoing invasive coronary angiography, 50% had no pathological obstructive lesions, which was triple the prevalence of nonobstructive coronary artery disease (CAD) in similarly referred men.^6^ Figure 1 illustrates the sex-stratified rates of ANOCA from large cohort studies (>500 participants) of male and female patients with a symptoms of ischemia who underwent an evaluation for ANOCA.^7^, ^8^, ^9^, ^10^, ^11^, ^12^, ^13^, ^14^ The difference in prevalence of ANOCA between men and women ranged from 2% to 32%, likely due to varying demographics, comorbidities, and diagnostic techniques.

Though traditional cardiovascular (CV) risk factors like hypertension, diabetes, and hyperlipidemia are associated with ANOCA, these patients tend to be younger with fewer CV risk factors.^1^

Importantly, patients diagnosed with ANOCA typically report a lower quality of life and persistent symptoms of angina with a resultant increase in health care utilization.^2^ They also report higher anxiety levels than those post-myocardial Infarction (MI), potentially due to lack of a definitive management plan after invasive coronary angiography.^15^

The most notable evidence of adverse outcomes in this population comes from the WISE trial, wherein 5-year annualized event rates for MACE (including MI, stroke, heart failure hospitalizations, and death) were found to be as high as 16%, and 7.9% in women with nonobstructive CAD and no CAD, respectively.^2^ In the same population, at 10-year follow-up, CV death or MI incidence remained high and occurred in 12.8% and 6.7% of women with nonobstructive CAD and no CAD, respectively.^6^

## Physiologic mechanisms

**Figure 2:**
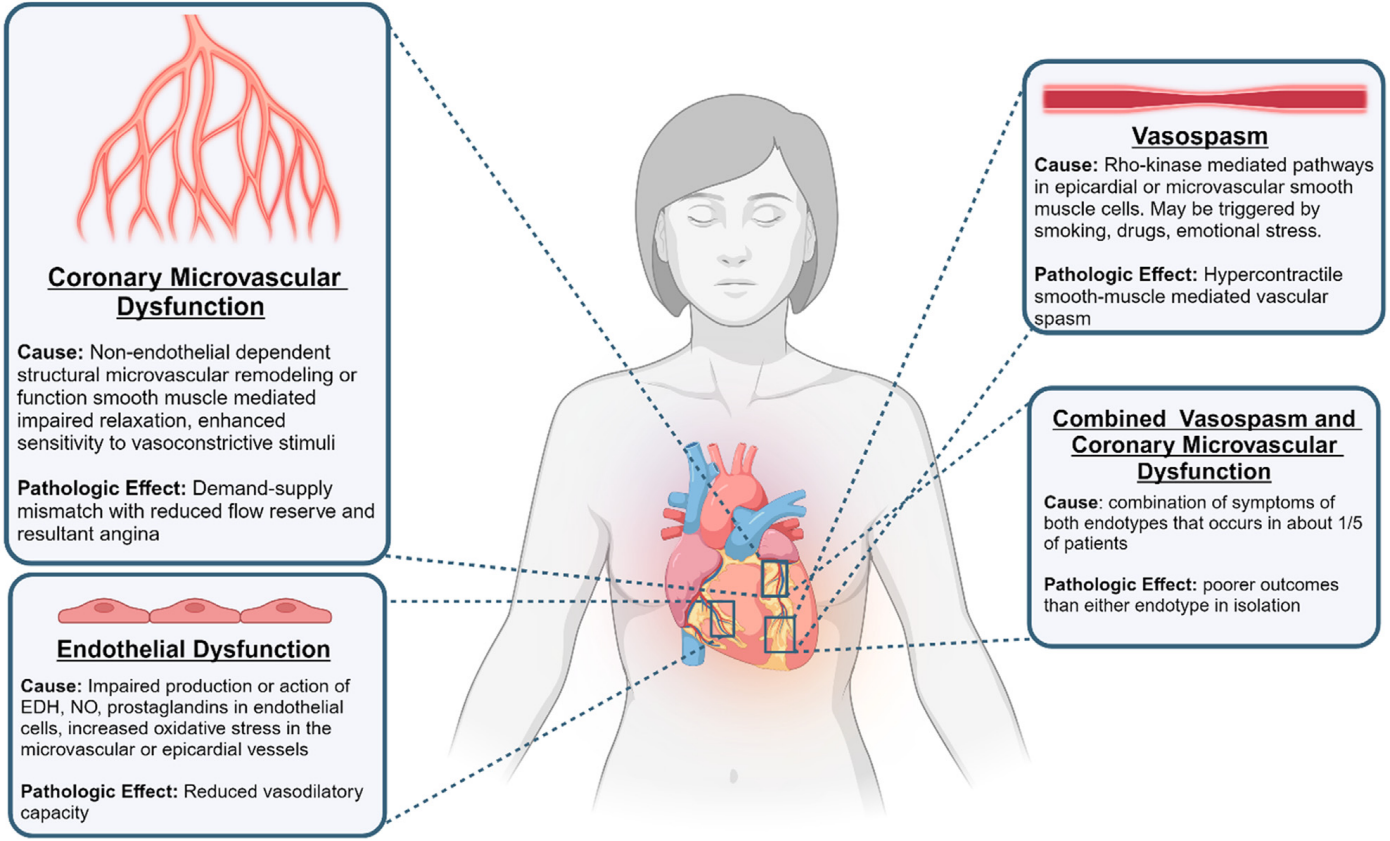
**Pathophysiologic Endotypes of ANOCA** Causes and pathologic effects of the four endotypes of ANOCA: 1) coronary microvascular dysfunction; 2) endothelial dysfunction; 3) vasospasm; and 4) combined epicardial vasospasm and microvascular dysfunction. ANOCA = angina with nonobstructive coronary arteries; EDH = endothelium-derived hyperpolarization; NO = nitric oxide.

Distinct pathophysiologic mechanisms, or endotypes, of impaired myocardial blood flow resulting in ANOCA have been described as below (Figure 2).^3^

### Coronary microvascular dysfunction

**Figure 3:**
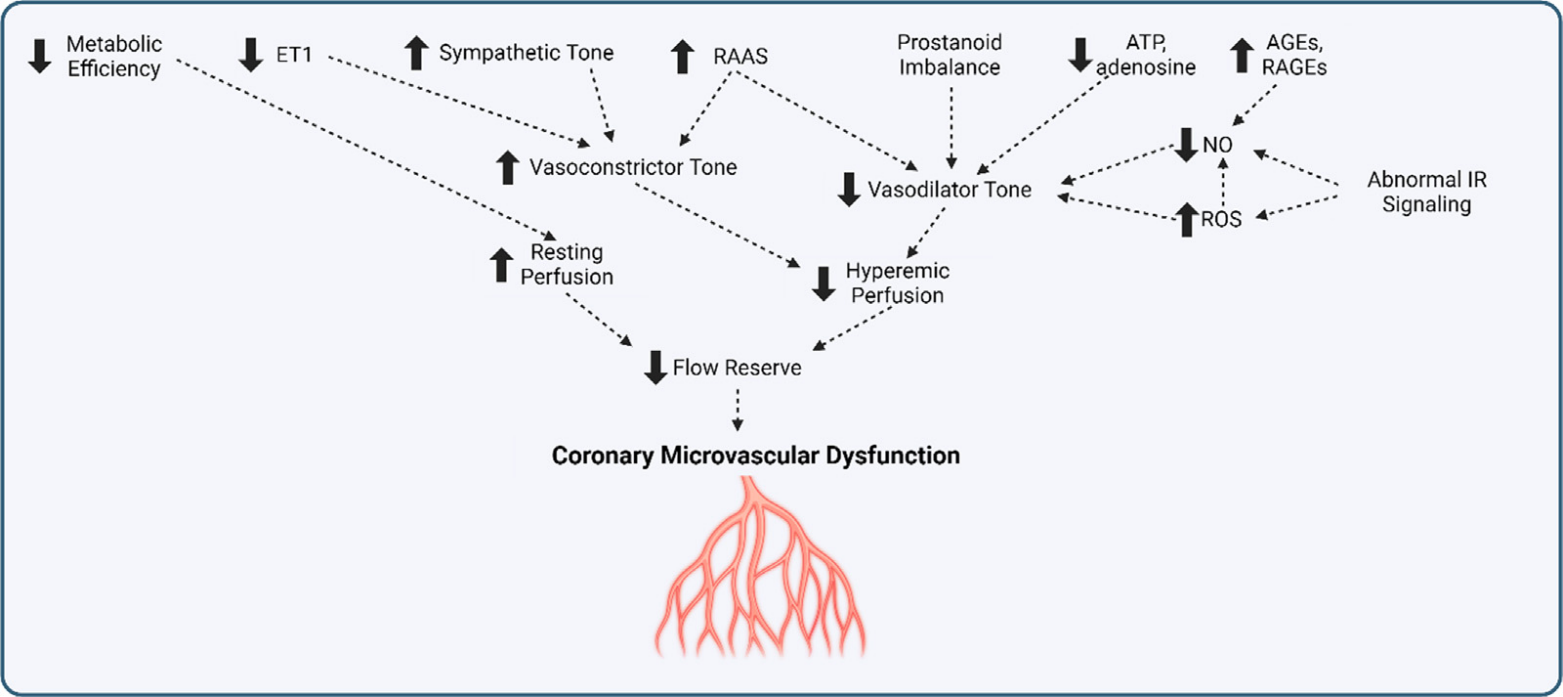
**Physiologic Mechanisms of Coronary Microvascular Dyfunction** Reduced flow reserve in coronary microvascular dysfunction is a result of reduced hyperemic blood flow or increased resting perfusion caused by multiple mechanisms. AGE = advanced glycosylation end products; ET1 = endothelin 1; NO = nitric oxide; RAAS = renin-angiotensin aldosterone system; RAGE = receptors of advanced glycosylation end products; ROS = reactive oxygen species.

The coronary microvasculature consists of prearterioles (100-400 μm), arterioles (40-100 μm), and capillaries. Prearterioles and arterioles are predominantly responsible for the resistance of the coronary circuit and the autoregulation of coronary blood flow (CBF) in situations of increased myocardial demand.^16^ Impaired functioning of the microvasculature results in a demand-supply mismatch with reduced flow reserve and resultant microvascular angina. In broad physiologic terms, this is a result of either reduced hyperemic perfusion or increased resting perfusion. Reduced hyperemic perfusion is characterized by low hyperemic and normal resting myocardial blood flow secondary to functional mechanisms. On the contrary, increased resting perfusion is a result of normal hyperemic, but elevated resting myocardial blood flow, signifying an increased metabolic demand and reduced metabolic efficiency due to probable abnormalities in oxygen delivery, utilization, or cellular metabolism (Figure 3).

By widely accepted definitions, the physiologic mechanisms of CMD are considered to be endothelium-independent. Functional mechanisms of CMD include abnormalities of the smooth muscle cells including impaired relaxation, enhanced sensitivity to vasoconstrictive stimuli, reduced sensitivity to vasodilators, or an abnormal increase in sympathetic tone.^16^ Sex differences in the pathophysiology of CMD exist as a result of sex hormone effects, autonomic regulation, and susceptibility to oxidative stress.^17^ Structural changes may result in microvascular remodeling and resultant luminal narrowing or obstruction. This can be a result of extrinsic vascular compression, vascular smooth muscle cell proliferation, perivascular fibrosis, vascular wall infiltration, microvascular inflammation, intravascular plugging, or insufficient increase in vascular density in response to myocyte hypertrophy.

### Endothelial dysfunction

A variety of molecular pathways within endothelial cells can impair the production or action of endothelial-derived relaxation factors such as nitric oxide (NO) and eicosonaoids, endothelium-derived hyperpolarization (EDH)-dependent vasodilation, or prostaglandins.^17^ These pathways can promote a state of high oxidative stress which inactivates vasodilator production and enhances the production of reactive oxygen species and other vasoconstrictive molecules such as endothelin-1 (ET1) and angiotensin II. Both result in an endothelial-dependent reduction in vasodilatory capacity of the microvasculature. It has been postulated that NO plays a larger role in epicardial vessels and EDH-mediated responses predominate in the microvasculature and their impairment results in epicardial or microvascular endothelial dysfunction, respectively.^17^ There may be an overlap between pathways mediating microvascular endothelial dysfunction and CMD. It is worth noting that EDH-mediated vasodilation has been shown to predominate in female microvasculature compared to men. The presence of CV disease (CVD) risk factors is associated with endothelial-dependent dysfunction. The association between mental stress and endothelial dysfunction with resultant myocardial ischemia in the absence of progressive CAD has also been described.^18^

### Vasospasm

This endotype is caused by a hypercontractile response of vascular smooth muscle cells to certain vasoconstrictor stimuli^3^ in either epicardial vessels or the microvasculature. It has been postulated that this exaggerated response typically involves pathways mediated by rho-kinase.^19^ Peaks in blood pressure, emotional stress, hyperventilation, smoking, and drugs are all potential vasospastic triggers.

### Combined vasospasm and coronary microvascular dysfunction

It is prognostically important to distinguish the combined endotype of vasospasm with CMD. A prospective study of patients (n = 187) undergoing invasive epicardial and microvascular function assessment showed that subjects with an elevated index of microcirculatory resistance (IMR) diagnostic of CMD and the presence of vasospasm had a significantly poorer long-term prognosis with a HR of 6.23 (95% CI: 1.21-118.48) for the cumulative incidence of cardiac death, nonfatal MI, or angina hospitalization, in comparison to subjects without coronary dysfunction or with the individual endotypes in isolation.^20^

Although not a discrete pathophysiologic subtype, abnormal cardiac nociception with enhanced pain sensitivity may contribute to symptoms of ANOCA. Prior studies have reported that patients with ANOCA have enhanced pain sensitivity with contrast injection during angiography, right ventricular pacing, and adenosine infusion, and pain is felt at a lower stimulus intensity compared to obstructive CAD patients.^21^ A case-control study of syndrome X patients (N = 8, 5 women) undergoing concurrent dobutamine stress echocardiography and dynamic positron emission tomography (PET) for cerebral blood flow showed distinct cortical activation patterns in patients with syndrome X suggesting that altered central neural handling of afferent stimuli from the heart may contribute to abnormal pain perception in these patients.^22^

## Diagnostic techniques

**Figure 4:**
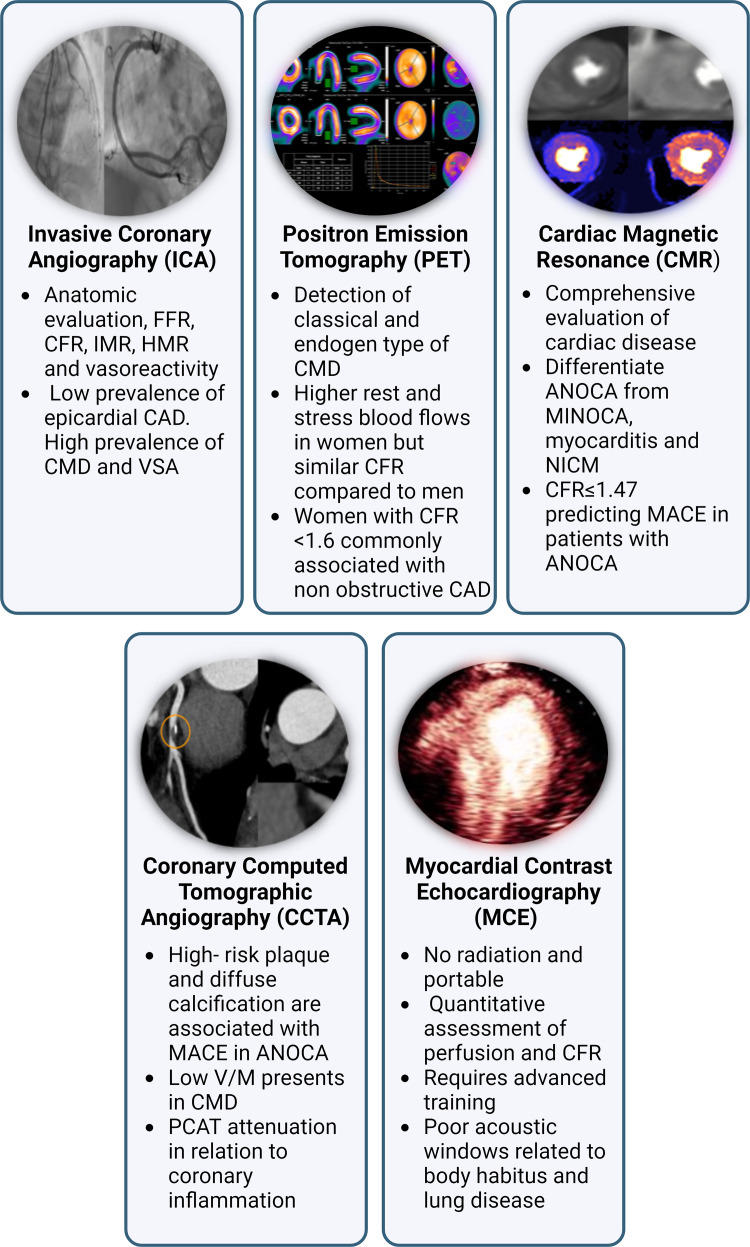
**Sex-Specific Diagnostic Considerations in ANOCA** Utility of various noninvasive and invasive diagnostic techniques in women with ANOCA. ANOCA = angina with nonobstructive coronary arteries; CAD = coronary artery disease; CFR = coronary flow reserve; CFVR = coronary flow velocity reserve; CMD = coronary microvascular dysfunction; FFR = fractional flow reserve; HMR = hyperemic microvascular resistance; IMR = index of microcirculatory resistance; MACE = major adverse cardiovascular events; MCE = myocardial contrast echocardiography; MFR = myocardial flow reserve; MINOCA = myocardial infarction with no obstructive coronary arteries; NICM = nonischemic cardiomyopathy; PCAT = pericoronary adipose tissue attenuation; V/M = coronary lumen volume to myocardial mass; VSA = vasospastic angina.

ANOCA may constitute nontraditional symptoms such as interscapular pain, nausea, indigestion, fatigue, weakness, dizziness, sleep disturbances, in addition to exertional chest discomfort or dyspnea, which could be under-recognized or dismissed as noncardiac in origin, particularly in young and middle-aged women.^23^ Therefore, a high index of suspicion during initial evaluation, and a sex-specific, endotype-defining diagnostic workflow is imperative. Figure 4 summarizes diagnostic modalities for ANOCA and their differential prognostic role in women.

### Invasive techniques

The stratified invasive diagnostic procedure for ANOCA combines direct evaluation of coronary vasomotor function with a guidewire, combined with pharmacologic vasoreactivity testing. A comprehensive approach toward invasive testing and interpretation has been described previously and is summarized here.^3^ Prior to functional coronary testing, temporary cessation of beta-blockers, CCBs, alpha-blockers, angiotensin-converting enzyme inhibitors (ACEI), and angiotensin receptor blockers is recommended.

First, conventional coronary angiography is performed to exclude anatomic obstructive CAD. Fractional flow reserve (FFR) is the ratio of mean distal coronary pressure to mean aortic pressure at maximal hyperemia during the infusion of adenosine with an FFR >0.8 indicative of no flow-limiting epicardial coronary disease.

Following this, coronary function can be assessed by a variety of metrics, all typically during the intracoronary infusion of adenosine (140 μg/kg/min) which causes nonendothelial-dependent vasodilation. The coronary flow reserve (CFR), representing both epicardial and microvascular function, can be calculated using thermodilution or a Doppler tipped guidewire, as the ratio of maximal to resting coronary flow. Cutoffs for abnormal CFR associated with prognostic impact have been <2.0 for thermodilution and <2.5 for Doppler techniques.^23^

The IMR represents microvascular blood flow.^10^ It is calculated as the product of distal coronary pressure (P_d_) at maximal hyperemia multiplied by the hyperemic mean transit time (T_mn_), which is determined using bolus thermodilution at maximal hyperemia. The principle behind IMR is analogous to Ohm’s law with the resistance across a microvascular bed, that is, the IMR, being equal to the pressure gradient across it divided by the hyperemic blood flow through it. As hyperemic blood flow is inversely proportional to mean transit time, and coronary venous pressure is negligible compared to Pd, the IMR is calculated as above. Increased IMR ≥25 is representative of microvascular dysfunction.

The last step involves vasoreactivity testing, to assess for vasospasm or endothelial-dependent CMD, with a graded infusion of intracoronary acetylcholine (ACh)—a trigger for endothelial NO synthase—being the most validated approach. An improvement in CBF of <50% or any reduction in CBF (calculated based on change in coronary diameter and flow velocity) with low-dose ACh suggests microvascular endothelial dysfunction.^3^ At higher doses of ACh, narrowing of the epicardial vessels from >0% to ≤90% suggests epicardial endothelial dysfunction, and a ≥90% reduction in epicardial coronary diameter, along with provocation of symptoms and ischemic electrocardiogram changes, is diagnostic of epicardial vasospasm.^23^ Microvascular spasm on ACh testing is suggested by anginal and ischemic electrocardiogram changes but without epicardial spasm.

There is often hesitation in the routine clinical performance of coronary function testing due to safety concerns, with widely varying practice patterns, all of which contributes to the underdiagnosis and inadequate treatment of specific ANOCA endotypes. The CorMica (Coronary Microvascular Angina) trial, which showed that these protocols were safe, cost-effective,^24^ and led to improved control of anginal symptoms by identifying endotypes^25^ should provide assurance and encouragement in this regard.

### Noninvasive techniques

Several noninvasive techniques provide ancillary information to invasive testing.

Coronary computed tomographic angiography (CCTA) is increasingly utilized in the initial evaluation of suspected ischemic symptoms. Coronary artery plaque burden and morphologic information obtained on CCTA is prognostic in women. A nested cohort within the PROMISE (Prospective Multicenter Imaging Study for Evaluation of chest pain) trial showed that the presence of high-risk plaque, defined as the presence of at least two adverse plaque features (positive remodeling, low-attenuation [<30 HU], spotty calcification, napkin ring sign) was a stronger predictor of MACE in women (n = 2,296) compared to men (n = 2,132) (adjusted HR: 2.41; 95% CI: 1.25-4.64 vs 1.4; 95% CI: 0.81-2.39) regardless of the severity of luminal stenosis.^26^ In the CORE320 (Combined Noninvasive Coronary Angiography and Myocardial Perfusion Imaging Using 320-detector Row Computed Tomography), presentation with INOCA as determined by CT angiography/CT perfusion was two-fold greater in women compared to men (12% vs 6%) and subjects with INOCA had a greater noncalcified atherosclerotic plaque burden and more prevalent high-risk plaque (positive remodeling and low-attenuation plaque volume) compared to those without ischemia.^27^

The ratio of total coronary lumen volume to myocardial mass (V/M) ratio has been demonstrated to be lower in patients with CMD.^28^ The ADVANCE (Assessing Diagnostic Value of Noninvasive FFRCT in Coronary Care) registry has described women as having a higher V/M ratio than men, driven by a lower myocardial mass.^28^ Therefore, a lower V/M ratio, particularly in women, should raise concern for CMD. CT myocardial perfusion can be performed in conjunction with the above techniques and has the potential to provide a comprehensive assessment coronary function, however needs further validation prior to widespread clinical use.

Pericoronary adipose tissue (PCAT) attenuation is another novel imaging biomarker of coronary inflammation and the vasoactive effects of adipokines. Coronary inflammation can exert paracrine effects on PCAT. The mean CT attenuation of PCAT can identify its composition, with less negative values signifying a more aqueous phase of PCAT indicative of vascular inflammation. An alternative technique of measuring PCAT using a proprietary fat attenuation index has been described. Higher PCAT has been associated with CMD as determined by lower coronary flow velocity reserve (CFVR) of the left anterior descending artery by stress echocardiography in patients with nonobstructive CAD.^29^ PCAT is a promising marker of identifying higher-risk patients with ANOCA and CMD, however needs further validation before widespread use.

The strength of PET and cardiac magnetic resonance (CMR) myocardial perfusion imaging lies in their ability to comprehensively quantify myocardial blood flow at stress and rest.^30^ Hyperemic/stress CBF is determined after the systemic administration of endothelial-independent vasodilators such as adenosine or regadenoson. The coronary or myocardial flow reserve is the ratio of stress to resting CBF. When obstructive epicardial stenosis has been ruled out and ANOCA is suspected, an abnormal global myocardial CFR indicates CMD.

PET is a well-validated technique of evaluating CBF and CFR, with a CFR <2.0 being widely accepted as the threshold defining CMD.^31^ Women with very low CFRs <1.6 more commonly have ANOCA and have a poorer prognosis compared to men.^32^ The determinants of low CFR can help distinguish whether CMD is due to reduced hyperemic perfusion or increased resting perfusion. The 2,023 Expert Panel Statement on PET reporting for CMD, terms the former as a “classical” and the latter as an “endogen” type of CMD.^31^ Women have been shown to have higher resting CBF compared to men,^32^ possibly related to sex differences in autonomic function.^17^ Therefore, the significance of “endogen type” CMD in women needs further elucidation. Though invaluable in the ANOCA diagnostic pathway, PET may be limited as a timely diagnostic modality due to availability and cost.

CMR myocardial perfusion imaging avoids ionizing radiation and provides an accurate assessment of microvascular function.^33^ In addition, it enables quantitation of cardiac chamber sizes, function, and evaluation of myocardial tissue characteristics which can aid in ruling out competing diagnoses. Recent advances in stress perfusion CMR now allow for the quantification of CBF and CFR^33^ which are prognostic and can identify patients at increased risk of death.^34^

Transthoracic echocardiography can be used in the assessment of ANOCA. One approach is to measure epicardial CFVR by transthoracic pulsed-wave spectral Doppler at rest and during vasodilator stress where CFVR <2.0 is considered abnormal.^35^^,^^36^ Limitations of this technique are that all three coronary arteries cannot be assessed in this manner in many patients, only coronary velocity rather than flow is measured, and poor acoustic windows may further impair quality, particularly in obese women. Myocardial contrast echocardiography is a noninvasive perfusion imaging technique that is quantitative and assesses spatial distribution of perfusion including transmural distribution without ionizing radiation. Myocardial contrast echocardiography perfusion imaging has also been used to assess populations with CMD in whether abnormal flow reserve is from increased resting flow or reduced hyperemic flow.^37^^,^^38^ Its main limitation is the specialized training needed for robust measurement of perfusion.

It is to be noted that patients with ANOCA may not exhibit definitive evidence of ischemia during evaluation with conventional diagnostic modalities such as treadmill exercise tests, stress echocardiography, or single-photon emission computed tomography. However, if no specific ANOCA endotype is identified on further stratified invasive investigation, noncardiac causes are to be considered. Notably in the CorMica trial of patients with ANOCA (N = 391), 11.3% patients had normal invasive coronary function after stratified testing and were diagnosed with noncardiac chest pain.^25^ In the absence of characteristic clinical features associated with specific noncardiac causes, noncardiac chest pain must be a diagnosis of exclusion.

## Incorporating precision medicine into anoca diagnosis and treatment

**Figure 5:**
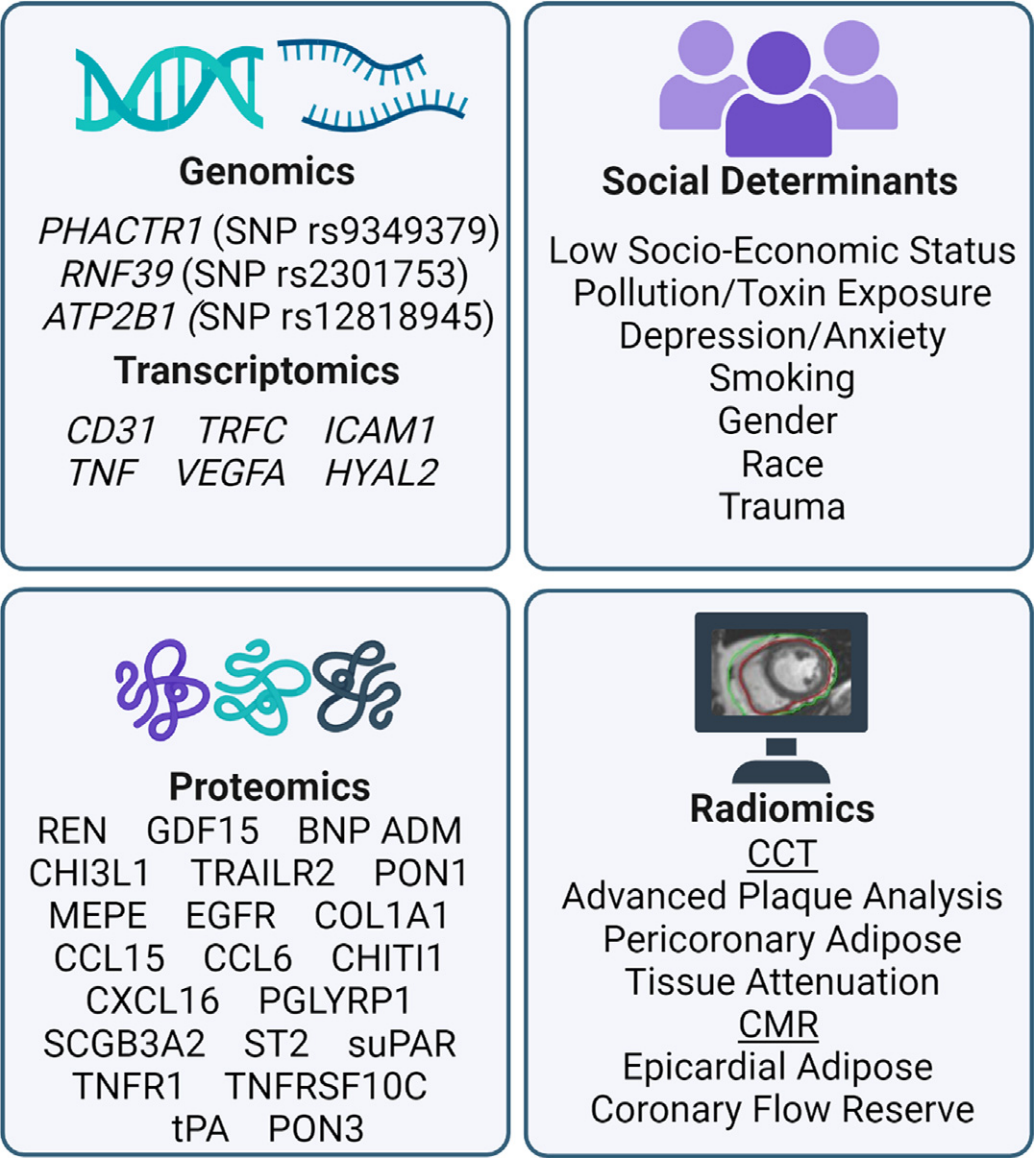
**Precision Medicine Considerations for ANOCA Diagnosis and Treatment** Examples of social determinants. Genomics, transcriptomics, proteomics, radiomics markers that have been associated with angina with nonobstructive coronary arteries (ANOCA).

Contemporary research is focused on defining a biological signature for ANOCA to diagnose it earlier, reduce invasive testing, predict risk, and determine tailored treatment options. Strategies involving radiomics, genomics, transcriptomics, proteomics, lipidomics, and consideration of social determinants are summarized (Figure 5).

### Radiomics

All the above CCTA-derived measures associated with ANOCA and CMD are predominantly determined qualitatively or semiquantitatively. Radiomics involves the automated extraction of multiple features describing a voxel, including its intensity/attenuation, textural, and spatial information, followed by machine learning-based selection of the most important features and development of a model that links these set of features to pertinent clinical parameters with the goal of precise quantification and prediction of disease.^39^ Radiomics-based models have been shown to perform better than CCTA alone at identifying plaque vulnerability.^40^ Radiomic assessment of PCAT has also performed better than PCAT attenuation-based models at identifying vascular inflammation in patients with acute coronary syndromes, compared to PCAT attenuation alone.^41^ Radiomics may enable more precise prognostication in patients with ANOCA and is a technique that merits further investigation.

### Genomics

The common genetic single-nucleotide polymorphism (SNP rs9349379-G) *PHACTR1* allele has been found to be associated with higher rates of CMD in a cohort of 391 patients.^42^ Additionally, Weng et al^43^ identified genetic variants in RNF39 (SNP rs2301753) and ATP2B1 (SNP rs12818945) to be correlated with higher instances of nonobstructive CAD in White women. While the specific mechanism of RNF39 is unknown, *PHACTR1* and *ATP2B1* have been established to play roles in NO signaling, which can impact CMD. Further pharmacogenomic and genetic biomarker studies geared toward early detection, predicting and monitoring therapeutic responses in ANOCA are necessary.

### Proteomics

Prescott et al^44^ determined more than 60 biomarkers that correlated with CFVR. Of these, the majority negatively correlated with CFVR, indicating an upregulation in patients with ANOCA (CFVR <2.5), including most strongly renin (REN), growth differentiation factor 15 (GDF15), brain natriuretic protein (BNP), N-terminal-proBNP (NT-proBNP), adrenomedullin (ADM), interleukin-6 (IL-6), chitinase-3-like protein (CHI3L1), death receptor 5 (TRAILR2), chemokine ligand 15 (CCL15), CCL16, chitinase 1 (CHIT1), C-X-C motif chemokine ligand 16 (CXCL16), galectin-4 (Gal4), matrix metalloproteinase 3 (MMP3), and tissue plasminogen activator (tPA) among others (Figure 5).^44^ The only protein identified from any studies that positively correlated with CFVR (indicating a downregulation in ANOCA patients) was PON3.^44^ Many of these proteins have known roles in immune/inflammation signaling or endothelial cellular adhesion/matrix remodeling as their mechanism for influencing CMD.

### Transcriptomics

There have been limited transcriptomics analyses of ANOCA. Bonanni et al^45^ investigated 38 patients diagnosed with either ANOCA (n = 18) or obstructive chronic coronary syndrome (n = 20) who underwent gene expression array analysis from peripheral blood mononuclear cells and identified lower expression of common immune markers including platelet and endothelial cell adhesion molecule 1 (*CD31*) and tumor necrosis factor in ANOCA patients. However, this study’s methods had several limitations, including the pooling of cDNAs for analysis, so the generalizability of their results is limited without follow-up studies.

### Lipidomics

Elevated plasma lipids, particularly oxidative lipoprotein epitopes that can reduce NO-mediated dilation, are recognized for their role in CMD and ANOCA.^46^ Microvascular dysfunction associated with hyperlipidemia can be reduced chronically with pharmacologic therapy such as statins, or acutely with plasmapheresis in those with familial hypercholesterolemia.^46^^,^^47^ Lower blood concentrations of long-chain fatty acid acylglycerols have been identified as a potential contributor to ANOCA, possibly through metabolic insufficiency for fatty acid oxidation and reduced metabolic efficiency.^48^ Abnormal ratios of certain eicosanoid metabolites of arachidonic acid have also been associated with ANOCA, consistent with their role in regulating vascular tone.^49^ Epicardial adipose tissue has been associated with ANOCA^50^ and is also known to be a source of specific lipidomic features that are thought to act in a paracrine fashion to promote CAD and possibly abnormal vasoactive tone.^51^

### Social determinants

Multiple social determinants of health (SDOH) adversely impact CV health in women. These include low socioeconomic status, neighborhood characteristics, limited access to health care, and environmental factors such as air pollution and toxic metal exposures.^52^ Indeed, a WISE substudy showed that a low socioeconomic status was associated with an increased risk of mortality among women presenting with chest pain, a majority of whom had no obstructive CAD.^53^ Inevitably, women of racial and ethnic minorities have a greater likelihood of experiencing adverse SDOH, in addition to discrimination, acculturation, and language barriers. Though data on race/ethnicity associations with ANOCA are sparse, a WISE substudy showed that Black women had higher rates of ANOCA compared to other populations, associated with greater MACE.^54^ Hispanic women have a higher prevalence of CVD risk factors such as diabetes, obesity, and metabolic syndrome. Disproportionately high rates of diabetes and premature CVD mortality are seen in Native American women.^52^ Lifetime traumatic exposures, which women of racial and socioeconomic minorities may be more prone to, have been linked to CVD. It has been shown that trauma-related dysregulation of the hypothalamic-pituitary-adrenal and sympathetic-adrenal-medullary systems can promote oxidative stress contributing to endothelial dysfunction.^55^

## Tailored therapeutic approaches and novel treatment options

Given the pathophysiologic and phenotypic heterogeneity of women with ANOCA, tailored therapeutic strategies are key. Most studies of ANOCA therapies have assessed anti-ischemic medications in relatively small, phenotypically diverse cohorts with short-term follow-up with modest results. However, the mechanistic insights provided by these studies lend themselves to being applied within a stratified framework. These have been described in detail prior reviews and are summarized here (Table 1).^3^

**Table 1:** Summary of the Current and Novel Therapeutic Strategies Targeting the Primary Endotypes of ANOCA

	Vasospasm	Coronary Microvascular Dysfunction	Endothelial Dysfunction
Lifestyle changes	Smoking cessation		
	Aerobic exercise		
	Weight loss		
First-line therapies	CCBs DHP (nifedipine, benidipine, amlodipine)	Beta-blockers (nebivolol, atenolol, metoprolol)	
	CCBs non-DHP (diltiazem, verapamil)	Statins (simvastatin, fluvastatin, pravastatin)	
	Statins (fluvastatin)	ACE inhibitors (enalapril, quinapril, ramipril)	
	Short-acting nitrates		
Second-line therapies	Cilostazol	CCBs (DHP/non-DHP)	SSRIs (escitalopram)
	Nicorandil	Aspirin	Trimetazidine)
	Fasudil	Ranolazine	Nicorandil
	Hormone therapy	Sildenafil	EECP
		Tricyclic antidepressants (imipramine, amitriptyline)	Hormone therapy
		Xanthine derivatives	
Novel targeted treatments			SGLT2 inhibitors
			Zibotentan
			GLP1 agonists

ACE = angiotensin-converting enzyme; ANOCA = angina with nonobstructive coronary arteries; CCBs = calcium-channel blockers; DHP = dihydropyridine; EECP = enhanced external counterpulsation; GLP1 = glucagon-like peptide-1; SGLT2 = sodium-glucose cotransporter-2.

### Anti-vasospastic therapies

CCBs (amlodipine, nifedipine, benidipine) have been shown to improve symptom frequency and prognosis in vasospastic angina (VSA) and are the only drug designated as first-line in these patients.^23^ Both vascular smooth muscle selective dihydropyridine CCBs and nondihydropyridine CCBs (diltiazem, verapamil) have been shown to be effective in the treatment of VSA, though the former have been more widely studied. Refractory VSA may warrant a combination of both classes of CCBs.^19^ Acute episodes of VSA often respond to short-acting nitrates. Insufficient evidence exists on the symptomatic or prognostic benefit of long-acting nitrates, and concerns of aggravating symptoms due to steal and increased MACE with tolerance from chronic nitrate use have been raised.^56^ Statins (fluvastatin) may benefit VSA by suppression of smooth muscle reactivity. Cilostazol, a phosphodiesterase-3 inhibitor, has been shown to improve VSA symptoms in multicenter RCTs. Nicorandil, a nitrate-like cGMP-mediated vasodilator and fasudil, a rho-kinase inhibitor, have been shown to improve VSA symptoms in multiple RCTs, however are not available in the United States. Smoking is a major trigger of VSA and cessation has been associated with a reduction in anginal symptoms and MACE.

### Therapies improving cmd and endothelial dysfunction

Though recognized as distinct endotypes, currently existing treatments are discussed together as they were studied in broader ANOCA populations with some overlap in their mechanisms of action and efficacy.

Beta-blockers, particularly nebivolol, improve endothelial function, symptom frequency, and exercise capacity in ANOCA. ACEIs which promote vasodilatory bradykinin and reduce vasoconstrictive angiotensin II (enalapril, quinapril) have been shown to improve anginal symptoms and CFR measurements in ANOCA. Statins improve NO bioavailability and studies of simvastatin, pravastatin, and fluvastatin have shown improvement in CFR. Low-dose aspirin has been shown to prevent oxidative stress and inhibits vasoconstrictive thromboxane A2. The multicenter WARRIOR trial is evaluating the effects of maximally tolerated statin, aspirin, and ACEI/angiotensin receptor blockers on MACE in women with ANOCA.^57^ Sildenafil, a phosphodiesterase-5 inhibitor, improves endothelial-dependent vasodilation and CFR.

Ranolazine may improve non-endothelial-dependent CMD and some studies have shown that patients of this endotype with lower baseline CFRs may derive greater benefit. Dipyridamole and xanthine derivatives (aminophylline, pentoxifylline, caffeine, theophylline) may benefit non-endothelial dependent CMD by redistributing CBF to ischemic regions where adenosine levels are greater. Studies of aminophylline in patients with CMD have demonstrated improvement in symptoms.

Selective serotonin receptor inhibitors such as escitalopram have been shown to improve angina in stable CAD patients with mental-stress-induced ischemia likely mediated by endothelial dysfunction and warrants further evaluation in ANOCA populations.^18^ Trimetazidine which has been shown to improve endothelial function and angina in ANOCA by altering cardiac myocyte metabolism and nicorandil, which improves CFR by NO-mediated pathways are not available in the U.S. low endogenous estrogen levels in postmenopausal women are associated with endothelial dysfunction and a WISE substudy showed that supplementation improved angina although recruitment was terminated prematurely after the adverse preliminary results of the Womens Health Initiative trial.^58^ However, recent long-term Womens Health Initiative outcomes showing no increased risk of mortality may enable further studies to be conducted into the role of hormone replacement in ANOCA. External enhanced counterpulsation has been shown to improve angina in patients with abnormal CFR with favorable effects postulated to be due to lowering vascular inflammation and improved endothelial function.^56^

Aerobic exercise, management of typical CV risk factors, and smoking cessation are all strongly recommended with all endotypes of ANOCA. Neuromodulating tricyclic antidepressants such as imipramine or amitryptiline may improve angina in ANOCA through anticholinergic and reduced norepinephrine uptake mediated visceral analgesic effects. Imipramine showed reduction in anginal symptoms in a placebo-controlled RCT.^2^ Similarly, spinal cord stimulation and transcutaneous electrical nerve stimulation improve angina and exercise tolerance in these patients. Relaxation therapy has been beneficial and may be a consideration in patients with stress-mediated symptoms where enhanced sympathetic vasoconstrictive responses are suspected.

### Novel targeted treatments

Cellular targets have been identified for more precision-based treatment of ANOCA and are being evaluated in ongoing trials.

ET1 is a potent vasoconstrictor of the coronary microvasculature, can cause regional impairment in myocardial perfusion, and is associated with endothelial-dependent CMD. Studies of ET1 receptor antagonists darusentan and atrasentan showed improvement in myocardial perfusion and endothelial function. The PRIZE (Precision Medicine with Zibotentan in Microvascular Angina) is an ongoing randomized, double-blind, placebo-controlled crossover trial will provide more definitive data on the role of ET1 receptor antagonists in improving symptoms, exercise tolerance, and myocardial perfusion.^59^

Several inflammatory markers are associated with endothelial dysfunction, and are promising targets of tailored therapies in ANOCA^60^: anakinra, an IL-1 receptor antagonist, has been shown to improve CFR in patients with rheumatoid arthritis; tocilizumab, an IL-6 receptor antagonist, improves measures of vascular stiffness in patients with rheumatoid arthritis; anti-tumor necrosis factor-α treatment has improved endothelial function in a variety of autoimmune disorders; cankinumab, an IL-1ꞵ antagonist has been linked to improved arterial stiffness. Glucagon-like peptide-1 receptor agonists have been shown to reduce CV endothelial dysfunction by reducing oxidative stress, promoting endothelial NO and reducing vascular inflammation in murine models,^61^ and have shown improvement in peripheral vascular endothelial dysfunction in diabetics.^62^ Indeed, glucagon-like peptide-1 receptor agonists are proving to be promising drug class warranting further study in ANOCA populations. Sodium-glucose cotransporter-2 inhibitors have demonstrated improvement in coronary microvascular function in animal models and improvement in endothelial function in diabetics.^63^^,^^64^ The impact of sodium-glucose cotransporter-2 inhibitors on CFR, symptoms, and quality of life in women with ANOCA is being studied in the SMILE (Effect of Dapagliflozin on Microvascular Function in Women With Symptoms of Coronary Artery Disease; NCT05762952) with an ancillary arm evaluating baseline and changes in inflammatory biomarkers with treatment.

## Importance of phenotyping in anoca and future directions

As highlighted, extensive heterogeneity exists in ANOCA at multiple levels including sex-specific differences in pathophysiology, a variety of pathophysiologic phenotypes, and a multitude of genetic factors and biomarkers associated with these phenotypes. The role of social determinants via epigenetic signaling adds another layer of complexity to the pathophysiology of ANOCA. These likely explain the incomplete responses to traditional antianginal treatments often seen with resultant increase in health care utilization, and poorer outcomes. Many knowledge gaps exist in several of these critical areas including the identification of high-risk phenotypes, the impact of SDOH and epigenetics on these phenotypes, and a strong evidence base to guide precise diagnostic workflow and treatment.

Addressing these disparities might be optimally achieved by employing a precision medicine-centric strategy. Ongoing research in the field will ideally involve machine learning-based network analysis of combined extensive genomic, transcriptomic, proteomic, lipidomics, and imaging phenotype data along with traditional endotype characterization to identify precise pathophysiology signatures, and consequently, effective targeted therapies. An ideal goal for ANOCA management would be the integration of stratified invasive/noninvasive testing, panomics, epigenetics, and social exposures into clinical decision-making pathways.

## Funding support and author disclosures

Dr Rodriguez Lozano is an iTHRIV Scholar. The iTHRIV Scholars Program is supported in part by the 10.13039/100006108National Center for Advancing Translational Sciences of the 10.13039/100000002National Institutes of Health under Award Numbers UL1TR003015 and KL2TR003016. Dr Hosadurg is supported by 5T32EB003841. Dr Kramer is supported by R01 HL075792. Dr Lindner is supported by R01-HL078610, R01-HL165422, and R01-HL130046 from the 10.13039/100000002NIH, and grant 18-18HCFBP-2-0009 from 10.13039/100000104NASA. Dr Villines has received salary support from Elucid Bioimaging, Inc, which is unrelated to this work. Dr Patel has received research support from G.E. Healthcare, Siemens Healthineers, CircleCVI, and Neosoft. Dr Sharma is supported by 10.13039/100000967AHA 979462. All other authors have reported that they have no relationships relevant to the contents of this paper to disclose. No authors received funding for their work on this manuscript.
